# Mechanisms of Intrinsic Tumor Resistance to Immunotherapy

**DOI:** 10.3390/ijms19051340

**Published:** 2018-05-02

**Authors:** John Rieth, Subbaya Subramanian

**Affiliations:** 1 Department of Surgery, University of Minnesota Medical School, 11-212 Moos Tower, Mayo Mail Code 195, 420 Delaware Street SE, Minneapolis, MN 55455, USA; jomrieth@iupui.edu; 2 Masonic Cancer Center, University of Minnesota, Minneapolis, MN 55455, USA

**Keywords:** cancer immunotherapy, resistance, oncogenes, signaling pathways

## Abstract

An increased understanding of the interactions between the immune system and tumors has opened the door to immunotherapy for cancer patients. Despite some success with checkpoint inhibitors including ipilimumab, pembrolizumab, and nivolumab, most cancer patients remain unresponsive to such immunotherapy, likely due to intrinsic tumor resistance. The mechanisms most likely involve reducing the quantity and/or quality of antitumor lymphocytes, which ultimately are driven by any number of developments: tumor mutations and adaptations, reduced neoantigen generation or expression, indoleamine 2,3-dioxygenase (IDO) overexpression, loss of phosphatase and tensin homologue (PTEN) expression, and overexpression of the Wnt–β-catenin pathway. Current work in immunotherapy continues to identify various tumor resistance mechanisms; future work is needed to develop adjuvant treatments that target those mechanisms, in order to improve the efficacy of immunotherapy and to expand its scope.

## 1. Introduction

Cancer immunology is an emerging and growing field with great therapeutic promise. Chemotherapy is relatively unselective and associated with rapid tumor resistance; by contrast, immunotherapy has the potential to provoke a lymphocyte response that can recognize neoantigens (which are generated by the constantly mutating cells of tumors), resulting in a more durable antitumor response.

Over the past few years, many strategies have been developed to modulate the immune system in an effort to optimize oncolytic properties while leaving healthy tissues intact. One such strategy is the use of checkpoint inhibitors, such as ipilimumab, an anticytotoxic T-lymphocyte-associated antigen 4 (CTLA-4) fully human monoclonal antibody, and pembrolizumab and nivolumab, both of which are programmed death 1 (PD-1) inhibitors. Such checkpoint inhibitors can prevent regulatory cellular markers from inhibiting the immune response. Currently, checkpoint inhibitors are the first-line treatments for patients with melanoma, renal cell carcinoma, and non-small cell lung cancer. Checkpoint inhibitors are also used for patients with refractory carcinomas (including head and neck tumors, bladder tumors, and Hodgkin’s lymphoma) and are now under consideration for patients with refractory sarcomas, among other tumors. As recently as 2017, the US Food and Drug Administration (FDA) approved pembrolizumab for patients whose tumors have mutations in mismatch repair or microsatellite instability.

Among the most promising future strategies in cancer immunotherapy is adoptive cell transfer. One example is chimeric antigen receptor (CAR) T-cell therapy, by which the patient’s own T cells are modified, ex vivo, by gene transfer techniques to create antitumor T cells [1]. In addition, dendritic cell vaccines [2] and immunotherapy with cytokine-induced killer (CIK) cells [3] are also under investigation.

Despite some success with immunotherapy in patients with untreated metastatic melanoma [4,5,6], squamous cell non-small cell lung carcinoma [7], and advanced renal cell carcinoma [8], most patients do not respond to immunotherapy, likely because of intrinsic tumor resistance. Intrinsic resistance involves the innate molecular qualities of the tumor that inhibit the immune antitumor response. Several such mechanisms have been proposed, including the reduction in antigenic expression and the diminishment of the quality and number of immune effector cells in the tumor microenvironment.

The other major type of mechanism is acquired resistance. Acquired resistance involves various mechanisms by which tumor cells develop resistance over the course of treatment, resulting in cancer progression despite an initial response to immunotherapy. Several such mechanisms have been proposed, including the loss of T cell function, the lack of T cell recognition due to immunoediting, and the development of escape mutation variant tumor cells [9,10,11].

Herein, we present an overview of several intrinsic mechanisms of resistance to current strategies of immunotherapy. Given a better understanding of such mechanisms, further strides can be made in evaluating and increasing the efficacy of immunotherapy, in expanding its use to patients with additional tumor types, and in developing adjuvant treatments.

## 2. Antitumor Immune Response

The antitumor immune response involves numerous cell types that collaborate to halt, or even eradicate, “foreign” tumor cells. Such collaborative cells, of either a lymphoid or myeloid lineage, are critical for causing direct oncolysis and for enhancing the activity of cytotoxic cells. Myeloid-derived cells, including dendritic cells, present foreign antigens to T cells and release cytokines that regulate the activities of cytotoxic cells.

**Figure 1:**
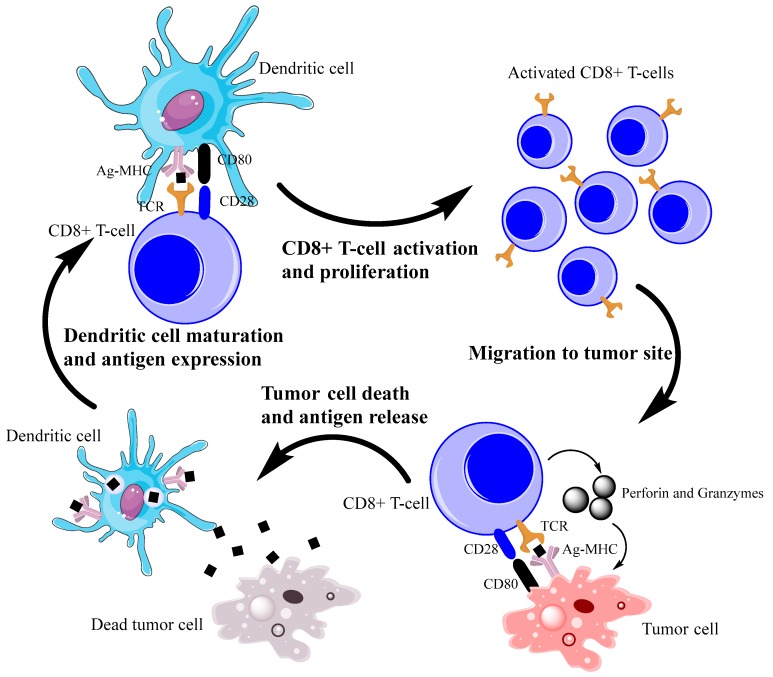
Simplified depiction of CD8^+^ T-cell antitumor immunity. CD8^+^ T cells are sensitized by dendritic cells via interactions between TCRs and antigen-bearing MHC I receptors, causing activation and proliferation of the CD8^+^ T cells. Then, CD8^+^ T cells migrate from the lymph node to the tumor site, targeting the antigen-bearing tumor cells for apoptosis via perforin and granzymes. The tumor cell releases antigens, which are captured by dendritic cells, which mature in the lymph nodes and cause further potentiation of T cells. Ag = antigen; MHC = major histocompatibility complex; TCR = T-cell antigen receptor.

Lymphocytes are a particularly important role in immunotherapy; depending on their class, they tend to have diverse functions. Some directly kill tumor cells and others release cytokines that promote cytotoxicity. Of the lymphocytes that directly kill, CD8^+^ cytotoxic T cells have been extensively studied. CD8^+^ T cells are antigen-specific: they are sensitized by dendritic cells via binding of CD8 and a tumor antigen-bearing major histocompatibility complex (MHC) I receptor. Once activated, CD8^+^ T cells recognize the presented antigens on the MHC I receptors of tumor cells, bind via a mechanism mediated by CD8-MHC I and CD28-B7, and release perforin and granzymes to induce caspase-mediated apoptosis. Additionally, CD8^+^ T cells release cytokines, such as interferon-gamma (IFN-γ), further potentiating the immune response (Figure 1). Tumor infiltration by CD8^+^ T cells correlates with favorable prognoses in patients with ovarian [12,13], colon [14,15], esophageal [16], or non-small cell lung [17] cancer.

CD4^+^ helper T cells tend to play a supportive role in the tumor microenvironment by coordinating the antitumor response. The two main types are T_H1_ cells (which promote cytotoxic responses) and T_H2_ cells (which promote hormonal immunity and upregulate the activity of eosinophils and neutrophils). Both types secrete cytokines to modulate the immune response: T_H1_ cells secrete interleukin (IL)-2 and IFN-γ; T_H2_ cells secrete IL-4, IL-5, IL-6, and IL-10, each of which invokes its own immune response [18]. Activation of CD4^+^ T cells (as with CD8^+^ cells) involves binding of CD4 receptors with an antigen-bearing MHC II receptor, along with CD28-B7 costimulation.

Previous studies have specifically indicated that high levels of T_H1_ infiltration were associated with favorable prognoses in patients with ovarian, colon, breast, renal, prostate, or cervical cancer [19], showing the critical role that T_H1_ cells play in antitumor immunity.

Clearly, T cells are important contributors to the antitumor immune response. So are natural killer (NK) cells, classically defined as CD3^−^CD56^+^ lymphocytes. Like T cells, NK cells are of a lymphoid lineage and have also demonstrated antitumor properties [20]. Unlike T cells, NK cells express no CD4 or CD8 markers, nor do they identify targets by antigen expression. Instead, NK cells recognize tumor cells by binding to proapoptotic cellular markers and by recognizing the absence of antiapoptotic markers on tumor cells, invoking cell lysis. As they recognize no antigens, NK cells do not require sensitization; thus, they contribute to innate immunity against tumor cells [21]. Like cytotoxic T cells, NK cells release granules of perforin and granzyme B to induce apoptosis by activating caspase. In addition, NK cells secrete numerous cytokines that enhance the cytotoxic immune response. They might also help detect tumor cells with reduced expression of MHC I receptors; such a reduction is a potential mechanism of resistance to T cells in several types of cancer.

## 3. Intrinsic Resistance Mechanisms

### 3.1. Reductions in Antigen Expression

Tumor-specific antigen expression plays an important role in the durability of the antitumor immune response by enhancing the development of antitumor-specific T cells. Recent studies have suggested that tumors with larger numbers of novel antigens tend to be more immunogenic, and are thus better targets for immunotherapy. For example, microsatellite-instable (MSI) colon cancers, which have mutations in the mismatch repair gene, generate 10 to 50 times more neoantigens than do microsatellite-stable (MSS) colon cancers without such mutations [22]. This increased antigen expression in patients with MSI (vs. MSS) colon cancer is associated with significantly increased T-cell infiltration and better prognoses.

Inherently low antigen expression is an intrinsic mechanism of resistance to immunotherapy. However, some types of tumors can develop acquired resistance to immunotherapy by selectively reducing—through an evolutionary process—expression of immunogenic, tumor-specific antigens. Through this process, the immune system eliminates the tumor cells with high antigen expression, sparing the variants with low antigen expression. The progeny of tumor cells with low antigen expression tend to have fewer immunologic targets, so the tumor can continue to grow. This process has been demonstrated in patients with metastatic melanoma [23].

Additionally, some evidence indicates that certain tumors can have reduced expression of MHC I receptors, inhibiting the presentation of tumor-derived antigens. Loss of MHC I expression has been suggested as a mechanism of resistance in patients with metastatic melanoma [24,25] and metastatic prostate cancer [26]. Reductions in MHC I expression have been associated with poor prognoses in patients with colorectal cancer as well [27]. Decreased expression of MHC I receptors and of transporters associated with antigen processing (TAPs), types 1 and 2, has been associated with progression of breast cancer [28]. Although loss of MHC I receptors can partially explain the loss of immunogenicity of certain tumors, it cannot fully account for tumor resistance, due to the actions of other oncolytic mechanisms that are independent of MHC I expression, such as oncolysis mediated by NK cells.

### 3.2. Alterations in Cell Receptor Expression

Another mode of tumor resistance to immunotherapy involves changes in the expression of cell receptors. Multiple cellular markers and receptors are expressed on both normal and malignant cells, either promoting or inhibiting cell lysis. Tumor-expressed markers, such as programmed death ligand 1 (PD-L1) [29], inhibit both the T-cell and NK cell response, whereas downregulation of Fas and tumor necrosis factor (TNF)-related apoptosis-inducing ligand (TRAIL) dampens lymphocyte-mediated tumor cell apoptosis [30]. Those markers are targets for current immunotherapy: PD-1 inhibitors prevent PD-1/PD-L1 binding; and CTLA-4 inhibitors prevent CTLA-4/CD-80 binding.

Reductions in MHC I chain-related gene A and B (MICA and MICB) have also been proposed as mechanisms of resistance that specifically prevent oncolysis mediated by NK cells via NKG2D [31]. MICA and MICB are stress-induced markers that bind to NKG2D on NK cells, thereby facilitating degranulation of NK cells and killing targeted tumor cells. Recently, MICA has been suggested as a potential efficacy predictor for future immunotherapy using cytokine-induced killer (CIK) cells. CIK cells are ex vivo generated lymphocytes that express a mixed T-cell and NK cell phenotype; they are later returned to the host and target malignant tumor cells. One clinical trial found better disease-free survival rates in gastric cancer patients with high MICA expression who were treated with CIK cells and adjuvant chemotherapy [32]. Similarly, another study found better survival rates in non-small cell lung cancer patients with elevated levels of MICA or MICB who were treated with cisplatin [33]. Unfortunately, in colorectal cancer patients, a reduction in MICA, in particular, has been found to be associated with poorer prognoses [34]; the reason could be proteolytic shedding of the cells, which can interact with NKG2D on NK cells, preventing the NK cells from interacting with tumor cells [35].

Currently, treatments are being considered that upregulate MICA, in hopes of potentiating cytotoxic lymphocytes. One example is all-trans retinoic acid (ATRA); when combined with CIK cell treatment in lung adenocarcinoma patients, ATRA significantly upregulates MICA and IL-2, enhancing the lysis activity of cytotoxic lymphocytes [36]. The definitive mechanism of ATRA in lung adenocarcinoma patients has yet to be elucidated. An earlier study in hepatocellular carcinoma patients found additional evidence of MICA upregulation and the resulting enhancement of cytotoxic lymphocyte function by ATRA [37]. Other treatments proposed for enhancing MICA expression include sodium butyrate, matrix metalloproteinase inhibitor III, and phenylarsine oxide, all of which were recently shown, in multiple myeloma cell lines, to enhance MICA expression and increase cytotoxicity [38]. Further investigation is needed to confirm the efficacy of MICA-enhancing treatment when combined with immunotherapy, as well as to test current MICA-enhancing treatment in additional tumors types with decreased MICA expression.

### 3.3. Alterations in CD28 Expression

Recently, CD28 has been extensively studied as a mechanism of resistance to current immunotherapy, and thus, as a target of novel strategies. CD28 is closely related to CTLA-4: both are immunoglobulin-related receptors that regulate immune function. Like CTLA-4, CD28 interacts with either CD80 or CD86. But CTLA-4 and CD28 perform opposing functions. CD28 is a costimulatory receptor, which activates a number of mechanisms that promote the immune response. Without CD28, exhausted T cells cannot proliferate and cannot perform normal lytic activity. Mechanisms activated by CD28 include the phosphatidylinositol 3-kinase pathway, Lck tyrosine kinase, IL-2-inducible family kinase Itk, protein kinase C, and adaptor proteins, such as GRB2 and GRB2-related adaptor downstream of Shc (GADS).

Those mechanisms culminate in the activation of transcription factors, such as nuclear factor-kappa B (NF-κB) and activator protein 1 (AP-1). The presence of those transcription factors is critical for IL-2 production and for T cell activation and survival; their absence results in T cell anergy. Additionally, recent studies suggest that CD28 could be associated with actin cytoskeleton remodeling via Vav-1, cofilin-1, and RLTPR. Because cytoskeleton remodeling is associated with full T cell receptor expression, CD28 could enhance T cell activation via this additional mechanism. CD28 expression is critical for T cell function in tumors treated with checkpoint therapy. Using mice treated with B7 antibodies and PD-L1 inhibitors, Kamphorst et al. demonstrated that CD28 inhibition resulted in progression of colon cancer, as did the suppression of T cell proliferation in CD28 knockout mice treated with PD-L1 inhibitors alone [39]. In the future, a potential strategy could be enhancing CD28 receptor expression to rescue exhausted T cells, propagating the antitumor immune response.

### 3.4. Alterations in Cellular Enzymes and Metabolic Pathways

Changes in antigen production and in receptor expression contribute directly to resistance to immunotherapy. Yet numerous other tumor cell alterations involving enzymatic activity and metabolism can also create changes within the tumor microenvironment, resulting in an inhibited response to immunotherapy. Four such mechanisms that have been proposed are induction of indoleamine 2,3-dioxygenase (IDO), loss of phosphatase and tensin homologue (PTEN) expression, deregulated expression of the Wnt–β-catenin pathway, and mutations in the interferon gamma (IFN-γ) Pathway.

#### 3.4.1. Induction of IDO

A heme-containing cytosolic enzyme, IDO, catalyzes tryptophan degradation in the kynurenine pathway, resulting in local tryptophan depletion. By modulating local tryptophan concentrations and releasing tryptophan metabolites, IDO assists in many critical biological functions, such as reducing the replication of viruses, bacteria, and parasites; preventing fetal rejection; and helping regulate the immune system. Because tryptophan degradation is a rate-limiting step in T cell progression through the G1 phase of the cell cycle, tryptophan depletion by IDO reduces T cell proliferation, thereby inhibiting T-cell activity against tumor cells [40]. IDO is associated with CTLA-4; according to in vitro studies, increased CTLA-4 levels have been shown to upregulate IDO in dendritic cells [41].

Several hematologic malignancies have been associated with an increase in functionally active IDO expression, including acute monocytic leukemia, acute lymphocytic leukemia, acute myeloid leukemia, and T-cell leukemia/lymphoma [42,43]. Additionally, previous studies have demonstrated an increase in functionally active IDO expression in various solid-tumor malignancies, such as breast cancer, colorectal cancer, endometrial cancer, gastric cancer, glioblastoma, gynecologic cancer, head and neck cancer, non-small cell lung cancer, small cell lung cancer, melanoma, mesothelioma, and pancreatic cancer, indicating a correlation [40,44].

IDO has also been suggested as a mechanism of resistance to CTLA-4 treatment, including ipilimumab. Holmgaard et al. demonstrated that mice with B16 melanoma were resistant to CTLA-4 treatment alone, but were susceptible to CTLA-4 treatment when it was combined with the IDO inhibitor 1-methyltryptophan (1MT) [45]. Holmgaard et al. also found similar results with anti-PD-1 treatment: IDO^−/−^ mice with B16 melanoma had a significantly reduced tumor burden and improved survival, suggesting that IDO inhibition could be a potential adjuvant to current immunotherapy [45].

Using the 4T1 orthotopic breast tumor mouse model, Monjazeb et al. recently showed that immunostimulatory treatments—including radiotherapy and the use of CpG oligodeoxynucleotide (a toll-like receptor 9 agonist)—was associated with a significant increase in cells expressing IDO (vs. controls), particularly in neoplastic epithelial cells. IDO mRNA expression was 3 to 5 times higher, suggesting that immunotherapy paradoxically increased the function of innate immunosuppressive pathways that could potentially limit efficacy. Then, when Monjazeb et al. [46] tested what they called “rebound immune suppression” by systemically treating the mice with “triple therapy”—1-methyl-D-tryptophan (D-1MT), an inhibitor of IDO, in combination with radiotherapy and CpG—they found that IDO activity decreased below the level in the controls. The “triple therapy” also significantly reduced tumor growth and improved survival. Finally, when Monjazeb et al. [46] tested the “triple therapy” in canines with spontaneous metastatic melanomas and sarcomas, they found a reduced tumor burden and improved survival (vs historical controls), suggesting that a canine model could be useful in the future for testing IDO-inhibiting strategies in humans with melanoma and sarcoma [46].

New therapeutic regimens that include IDO antagonists are under study in current clinical trials, most notably epacadostat and indoximod. Unfortunately, those regimens often have significant side effects. Epacadostat is associated with grade 3 and 4 side effects for abdominal pain, hypokalemia, and fatigue; indoximod, with grade 1 fatigue and grade 2 hypophysitis [47,48]. IDO therapy could be combined with other treatments, including checkpoint inhibitors, to maximize therapeutic response [49].

#### 3.4.2. Loss of PTEN Expression

Loss of PTEN, a tumor suppressor, has also been implicated in resistance to immunotherapy. A lipid phosphatase, PTEN regulates intracellular PI3K signaling, thereby reducing cellular proliferation and survival. Loss of PTEN expression results in increased tumor cell survival and enhanced tumor growth; additionally, tumors with cells lacking PTEN also tend to be poorly immunogenic. Several studies of tissue specimens of glioblastoma demonstrated that T cells more effectively lysed tumor cells with wild-type *PTEN* and were less effective in lysing tumor cells with mutant *PTEN*: the decrease in lysis was associated with an increase in B7-H1 cell receptor expression. *PTEN* mutant cells transfected with wild-type *PTEN* had increased susceptibility to lysis induced by T cells—further evidence of the importance of PTEN function in immunotherapy [50].

In experimental models of mice with melanomas treated with adoptive T-cell therapy, Peng et al. showed that silencing *PTEN* decreased T-cell activity against tumor cells, both in vitro and in vivo, demonstrating resistance to T-cell-mediated killing of tumor cells.

Clinical research by Peng et al. showed that PTEN was correlated with decreased tumor infiltration, decreased function of T cells, and poorer outcomes in human melanoma patients treated with checkpoint therapy. The mechanism of such resistance in PTEN-negative tumors is currently unclear, although errors in PTEN might confer resistance to tumors via the release of anti-inflammatory cytokines, such as CCL2 and vascular endothelial growth factor (VEGF), resulting in reduced tumor cell infiltration by CD8^+^ T cells [51].

Resistance to PTEN-associated checkpoint therapy has also been observed in patients with metastatic uterine leiomyosarcoma [52] or glioblastoma [53]. More research is needed to further characterize the mechanism of this resistance pattern, and to determine whether PTEN is an appropriate target in future immunotherapy.

#### 3.4.3. Deregulated Expression of the Wnt–β-Catenin Pathway

The Wnt–β-catenin pathway plays a critical role in oncogenesis and contributes to tumor resistance to oncolysis mediated by the immune system. The binding of Wnt to its receptor results in an accumulation of intracellular β-catenin, promoting transcription; in contrast, a lack of Wnt–receptor complex binding results in β-catenin proteolysis. In an experimental melanoma study, Spranger et al. demonstrated that tumor-intrinsic β-catenin activation prevented T cell priming and infiltration into the tumor microenvironment via defection of CD103^+^ dendritic cell recruitment. They attributed the mechanism of inhibited dendritic cell infiltration to, in part, a reduction in the production of the chemokine CCL4 by Wnt–β-catenin activation [54].

CCL4 is a critical molecular signaling molecule that serves as a chemoattractant molecule for NK cells, monocytes, and other components of the immune system [55]. Previous studies have demonstrated that CCL4 expression is associated with an improved response to immunotherapy, including ipilimumab, in melanoma [56]. The prevention of dendritic cell migration to the tumor microenvironment also prevents antigen presentation to T cells, thereby inhibiting T cell development and stunting oncolysis of tumor cells.

### 3.5. Activation of the IFN-γ Pathway

Another key element of the immune response is the IFN-γ pathway, which can serve as a mechanism of resistance to immunotherapy. IFN-γ is a cytokine produced and released by activated T cells and antigen-presenting cells. It acts as a chemical messenger via a mechanism mediated by a Janus-activated kinase (JAK). Several downstream cascades are thus regulated, classically through interaction of JAK1 or 2 with signal transducer and activator of transcription proteins (STATs). The results include expression of antigen-presenting molecules, increased recruitment of cytotoxic cells, and reduced proliferation of tumor cells [57]. Loss of function of this critical component of the immune response prevents a significant response to checkpoint therapy. In human patients with melanoma, responders (vs nonresponders) to ipilimumab have been found to be much more likely to have mutations involving IFN-γ. Likewise, experimental models of knockout mice demonstrated that the absence of the IFN-γ pathway resulted in increased tumor growth and resistance to CTLA-4 therapy [58]. Similarly, human patients with metastatic melanoma and metastatic colon carcinoma who lost IFN-γ function, due to JAK mutations, had innate resistance to PD-1 checkpoint therapy [59].

## 4. Discussion

In recent years, the field of oncoimmunology has fostered an increased understanding of cancer biology, leading to the development of several therapies that have shown great potential to invoke durable responses against cancer. Despite the demonstrated successes of immunotherapy, most patients do not respond. The proposed mechanisms have included a lack of immune cell infiltration, poor antigen expression, and tumor-mediated silencing of the immune system via cytokine release. However, additional mechanisms of resistance continue to be discovered, further elucidating the complex interactions between cancer and the immune system [9,60,61]. The predominant mechanisms are summarized in Table 1.

These mechanisms of resistance not only define the outcomes and limits of current immunotherapy, but also point to future adjuvant treatments to facilitate antitumor immunity. It is already established that radiation of tumors can increase the generation of neoantigens for immunotherapy [67,68,69]. Certain chemotherapy agents, such as gemcitabine [70], have also been noted to increase lymphocyte infiltration in the tumor microenvironment. New treatment strategies include tumor vaccinations, as well as gene therapy as a method of introducing novel antigen targets into tumor cells, in addition to directly causing tumor cell death [71,72,73]. Treatments that target other noted mechanisms of resistance, including IDO overexpression, are now undergoing clinical trials [48].

One exciting new avenue of study is epigenetic research of the tumor microenvironment. Epigenetic markers, including nucleotide methylation and posttranslational histone modification, can make DNA either more or less accessible for transcription, thereby either activating or silencing transcription. By regulating transcription, epigenetic markers can either prime or inhibit the immune response in the tumor microenvironment; they are also thought to contribute to reduced immunological expression in resistant tumors. Recent research has demonstrated that epigenetic markers can be targeted by regulating the proteins that facilitate immunological changes, including DNA methyltransferase (DNMT) and histone deacetylase (HDAC). For example, HDAC inhibitors have been shown to enhance T cell function and survival [74]. Currently, multiple clinical trials of combination treatment with epigenetic drugs and immunotherapy are enrolling patients with various cancers, including non-small cell lung cancer, breast cancer, and melanoma [75].

Future studies should build on those trials and seek additional targets that might amplify the antitumor immune response, with the ultimate goal of increasing the rates of lasting responses to immunotherapy. Molecular targeted therapy is an intriguing solution that could help overcome the intrinsic resistance to immunotherapy. It works by selectively blocking essential biochemical pathways or by inhibiting mutant proteins that are crucial for tumor cell growth and survival, such as tyrosine kinases. So far, molecular targeted therapy has invoked some dramatic, but typically short-term, responses. By contrast, immunotherapy tends to invoke lasting responses, but only in a few patients with certain cancers [76]. The different strengths and weaknesses of molecular targeted therapy and immunotherapy suggest that they could play complementary roles. Molecular targeted therapy could inhibit the immunosuppressive environment generated by tumor cells, permitting lymphocyte infiltration of the tumor in order to generate an effective immune response.

Of course, future research should continue to focus on expanding the scope of checkpoint immunotherapy to additional tumor types, including sarcoma. Immunotherapy could potentially be used to treat sarcoma patients, as demonstrated by reports of extensive T-cell infiltration into sarcoma tumors. Several different sarcoma tumors, including dedifferentiated chondrosarcoma [77], infantile fibrosarcoma [78], synovial sarcoma [79], and osteosarcoma [80], have been evaluated for T-cell expression and immune checkpoint expression.

In conclusion, immunotherapy is a rapidly expanding field that has transformed oncology. The use of checkpoint inhibitors has expanded to treat patients with numerous types of cancer, including melanoma, renal cell carcinoma, and non-small cell lung cancer; it has also been approved for patients whose tumors have mutations in mismatch repair (e.g., MSI colon cancers. Currently, the efficacy of immunotherapy is limited by mechanisms of resistance. By further exploring those mechanisms and developing strategies to counter them, the scope and success of immunotherapy could be significantly enhanced.

## Figures and Tables

**Table 1:** Mechanisms of resistance to immunotherapy.

Mechanism	Examples of Associated Cancers	Citation
Reduced neoantigen generation	colorectal, kidney clear cell	Rooney et al. (2015) [62]
Reduced Fas expression	melanoma	Bullani et al. (2002) [63]
Reduced TRAIL expression	melanoma	Nguyen et al. (2001) [64]
Reduced MICA or MICB expression	colorectal, gastric	Salih et al. (2002) [35]
Reduced MHC I expression	melanoma, lung, breast, renal, prostate, bladder	Campoli et al. (2008) [65]
Induction of IDO	acute myeloid leukemia, colorectal, endometrial, small cell lung, melanoma, ovarian	Moon et al. (2015) [66]
Loss of PTEN expression	melanoma	Peng et al. (2016) [51]
Deregulation of Wnt–β-catenin pathway	melanoma	Spranger et al. (2015) [54]
Loss of function of IFN-γ pathway	melanoma, colorectal	Shin et al. (2017) [59]

IDO = indoleamine 2,3-dioxygenase; IFN-γ = interferon-gamma; MHC = major histocompatibility complex; PTEN = phosphatase and tensin homologue; TRAIL = tumor necrosis factor (TNF)-related apoptosis-inducing ligand.
